# Restoring Vision through “Project Prakash”: The Opportunities for Merging Science and Service

**DOI:** 10.1371/journal.pbio.1001741

**Published:** 2013-12-17

**Authors:** Pawan Sinha, Garga Chatterjee, Tapan Gandhi, Amy Kalia

**Affiliations:** Department of Brain and Cognitive Sciences, Massachusetts Institute of Technology, Cambridge, Massachusetts, United States of America

## Abstract

By treating curably blind children in India, “Project Prakash” brings sight to children of different ages, offering insights into how their brains adapt to enable them to see. The project's experience highlights the benefits of merging basic research with societal service.

“So how does this help society?” is a question we are often asked as scientists. The lack of immediate and tangible results cannot be held against a scientific project but statements of future promise in broad and inchoate terms can sometimes pass the benefit-buck indefinitely. There is no incentive against over-stating the benefits, especially when they are hypothetical and lie in the distant future. Few scientists will say their science is not designed to serve society. Yet the proliferation of “potential benefits” in grant proposals and the Discussion sections of research papers, in the absence of tangible translations, can make the service element of science seem like a clichéd ritual. Its repetition hollows out its meaning, breeding cynicism about the idea that basic science can be of service.

Expanding human knowledge can be tied intrinsically to betterment of the human condition. Our personal desire to be good Samaritans and our professional desire to be good students and researchers are not necessarily at odds. This aspiration to merge science and service can only come to fruition, however, if we actively identify opportunities that merge the two—the kind of science that necessitates service, but not at the cost of trivializing either. Here, we present Project Prakash as a prototype of what such initiatives might look like.

## Project Prakash

**Figure 1:**
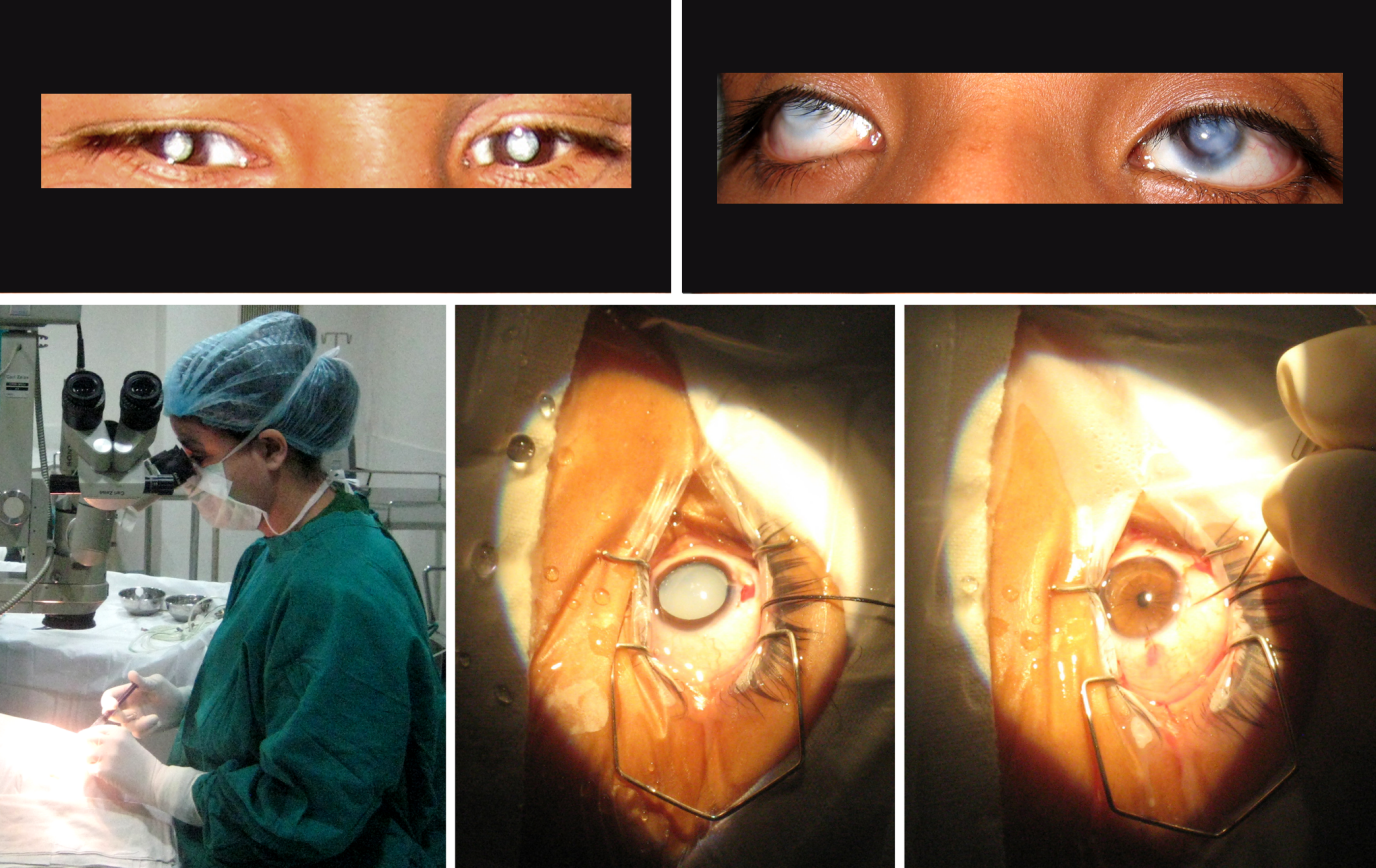
Top row: Two major kinds of childhood blindness in India. The left panel shows cataracts, and the right shows corneal opacities. Many cases of pediatric cataracts are congenital. The lower row shows, from left to right, cataract surgery in progress, a dilated pupil revealing a dense cataract, and the eye after excision of the cataract and implantation of an intra-ocular lens.

The Prakash initiative [1] identifies congenitally but curably blind children in India who have so far remained untreated due to poor medical access. Figure 1 shows two major kinds of treatable childhood blindness in India—cataracts and corneal opacities. By providing such children corrective surgery (Figure 1, lower panels), Project Prakash is creating a population of children across a wide age range who are just learning how to see. Longitudinal studies of these unique children offer insights into how they develop visual skills. This approach combines the best of both worlds: it directly addresses the pressing humanitarian need to treat curably blind children and, in the process, illuminates fundamental questions regarding brain plasticity and learning. Hence the name “Prakash,” the Sanskrit word for light. To date, Project Prakash has screened over 40,000 children and provided surgical treatment to over 400 of them as well as non-surgical care to over 1,400.

### Scientific Outcomes from Project Prakash

The Prakash studies have yielded evidence of significant adverse effects of congenital blindness on some aspects of post-operative vision, but also impressive visual skill acquisition by those who gain their sight even after several years of blindness [2]–[5]. We have investigated how patients acquire many aspects of visual function, including low-level attributes such as acuity and contrast sensitivity as well as high-level functions such as object and face recognition and spatial imagery. This work has also demonstrated how motion information plays a crucial role in parsing the world into distinct objects and, more importantly, in helping the visual system learn heuristics that can be used even with static images. The results have guided our studies of normally sighted individuals, as well as our computational modeling efforts [6]–[8]. Complementing our behavioral studies, we have begun to employ non-invasive brain imaging technology, specifically functional magnetic resonance imaging (fMRI), to examine the kinds of cortical changes that accompany the very initial stages of human sensory development. These neuroimaging studies so far have revealed that the onset of patterned visual information results in rapid modification of the visual cortex even in individuals who have been congenitally blind for the first two decades of their lives [9].

Recently, Project Prakash also helped answer a long-standing philosophical question about the mind. The onset of sight challenges the brain to correlate visual information with that from other senses such as touch and audition. What is the genesis of such cross-modal mapping? Is the ability to relate information from distinct senses available innately, or is this something that needs to be learned through experience? This fundamental question, which was framed over three centuries ago by philosophers John Locke and William Molyneux, is relevant to understanding how the brain encodes an object's shape and also the “nature *versus* nurture” debate [10]–[12]. Project Prakash has finally allowed us to provide an answer to this tantalizing question [5]. Immediately after the onset of sight, the Prakash children could not recognize by sight an object they had felt with their hands (i.e., there was no evidence of an innate link between vision and touch), however, they became proficient in as little as a week. The development of this ability points to the existence of rapid learning processes for detecting relationships across senses.

Our findings paint a picture of a brain that remains impressively adaptable well into life and that can reorganize itself quite rapidly to allow a newly sighted child to make use of the novel sensory information received from its eyes. These early findings provide a launch pad for studies that are sure to enrich our understanding of the processes by which we acquire our diverse visual abilities.

### Service Outcomes from Project Prakash

Besides providing novel insights regarding basic questions in experimental and theoretical neuroscience, this project is also yielding more pragmatic benefits.

#### Ophthalmic care for poor children

Many poor families, especially those in rural communities, lack access to ophthalmic care. The outreach efforts of our project have allowed us to bring the benefits of modern medicine to several of the most medically neglected and financially impoverished communities in India.

#### Enhancing social acceptability and awareness of childhood blindness

In Indian society, childhood blindness carries grave stigma and is poorly understood, leading to superstitions, dangerous non-medical “remedies,” or, often, no treatment at all for the affected children. Project Prakash has begun to increase awareness in India of this condition and the available treatment options, removing misconceptions from parents' minds and inducing them to seek proper treatment for their children. Furthermore, by highlighting the problem of childhood blindness in prominent international forums and journals, the project is helping to catalyze further initiatives by philanthropic and government agencies to counter the problem of childhood blindness in India and elsewhere.

#### Facilitating education

For the formerly blind children, the onset of sight makes it possible to enter the educational mainstream and thus to better their prospects for employment and independence. The reports, audio/visual material, and papers on basic issues in visual neuroscience generated by Project Prakash also aid education in this domain.

The magnitude of the problem of childhood blindness in the developing world is daunting, and the challenge of unraveling brain mechanisms of visual learning is amongst the hardest in science. But, we are encouraged that Project Prakash has begun to serve as a nucleus for bringing together the resources, expertise, and commitment needed to mount appropriate responses. In undertaking this effort, we have a unique and unprecedented opportunity to address issues of great humanitarian, health, and scientific significance.

Aligning service and science can be a powerful approach for our community. Initiatives like Project Prakash can provide resources and directions to talented people, channeling their skills, passions, and scientific abilities to solve important problems in society. In Box 1, we outline some other examples akin to Project Prakash, where biomedical research might be combined with service to the community. Such approaches have the effect of bringing science and society closer—providing the public with a strong reason to continue funding science. Developing projects along the lines of Project Prakash requires actively searching for scientific questions whose answers require interventions that improve lives, society, or the environment as a constitutive part of the research protocol. Obviously, not all scientific questions can meet this criterion and, when they can, there are potential hurdles to overcome in combining science and service (Box 2). Implementing this model may well require more effort than usual to achieve successful outcomes; however, the dramatic improvements in the lives of those who are helped, the scientific advances, as well as the personal satisfaction make the effort worthwhile.

**Box 1. Other “Prakash” Opportunities**

We believe that Project Prakash is just one instance of many compelling opportunities to merge science and service. These “Prakash” Opportunities promise to bring light into people's lives and simultaneously illuminate fundamental questions in science. Here are a few examples.

*Educating illiterate individuals and seeing effects on brain:* A third of the global population is illiterate [13]. Illiteracy affects not only the individual but also society. Providing education to illiterate individuals will help improve their life prospects while also presenting an opportunity to study changes in various aspects of cognition and their neural substrates.

*Providing treatments for other childhood disabilities and studying outcomes:* The prevalence of childhood disability has increased significantly in the last few decades [14]. Rudimentary interventions are available for some disabilities, such as sensory impairments, speech impediments, epilepsy, and autism, which improve children's ability to interact with their world, Nevertheless, they are far from perfect. Providing children the best available treatments is a great opportunity to study treatment outcomes as well as learning and plasticity mechanisms in the brain. Such studies can help determine how the interventions themselves can be improved.

*Feeding malnourished children and probing effects on brain structure and function:* Severe malnourishment is responsible for the deaths of 5 million children every year [15]. Infants born underweight are at risk for learning disabilities, mental retardation, blindness, and even premature death [16]. By providing nutrition to chronically malnourished individuals, we have the opportunity to improve their lives while also examining how the brain responds to this intervention at different points in the developmental trajectory. For instance, imaging techniques can be used to determine if improved nutrition results in changes in brain volume and connectivity. The inclusion of scientific assays can, in turn, enhance the ability to monitor the effectiveness of nutritional interventions.

*Providing medical screening and detecting low-penetrance disease susceptibility genes:* Many conditions, like various kinds of cancer, are likely to arise from several low-penetrance genes rather than a single high-penetrance mutation [17],[18]. The discovery of these genes requires large numbers of well-characterized patients. This scientific need is perfectly complementary to the humanitarian need for increased disease screening in densely populated developing nations. The large family structures in many of these societies, and hence the availability of genetic data across many relatives, can greatly facilitate gene discovery.

**Box 2. Notes of Caution**

As appealing as it sounds to combine science with humanitarian efforts, there are potential pitfalls to this approach. These include implementational challenges as well as broader issues of ensuring that research creativity is not stifled.

*Financial support*: A project based in a foreign country, whose immediate beneficiaries are individuals of that country, might be seen as beyond the ambit of a national funding body. We find that, on the contrary, funding agencies are very supportive of Project Prakash because they see that the results are likely to transcend national boundaries. More generally, funding risks are ameliorated if the anticipated scientific gains are likely to be significant and not geographically specific.

*Support from the research community*: A project with a service component might be seen as a distraction from our obligations to publish papers, obtain grants, and fulfill the requirements for tenure. Our experience with Project Prakash has been overwhelmingly positive, however: researchers generally share a desire to affect people's lives, even though this motivator can be lost in the shuffle of grant deadlines and paper submissions. We feel that there is widespread support for research programs tailored to a humanitarian cause that also addresses important scientific questions.

*Support from society at large*: A project that merges science and service risks being misinterpreted as exploitative or disrespectful of cultural and religious traditions, especially in developing countries where there is greater resistance to science-based interventions due to competing religious, cultural, or community beliefs. This very real danger must be countered with clear communication between scientists and society. In Project Prakash, we find that counseling parents, a frank description of the project's objectives, and a few local non-scientist champions whom the patient population can relate to, are often necessary and sufficient to address this concern.

*Maintaining high quality of research*: Many researchers can attest to the challenges of working with children and patients. For instance, densely sampled longitudinal studies are logistically complex. The histories of the participants are highly variable, sometimes making group level analyses difficult. Such hurdles may be exacerbated in a developing country but can provide a useful spur to designing experimental protocols that are resilient to the circumstances. Some unavoidable experimental compromises may be tolerable as long as they are accurately reported and do not hinder the interpretation of the results.

*Draining resources from other deserving avenues of research*: The desire to tie research to societal benefits should not diminish our ability to pursue more “blue-sky” projects. The scientific value of any project must be evaluated dispassionately. Are the likely scientific advances significant enough to merit the investment into the project? If the answer is affirmative, then all else being equal, a project that has a simultaneous beneficial impact on society may be more deserving of support than one that does not.

*Maintaining ethical standards*: Linking the provision of service to scientific gains may result in a conflict of interest, transforming service into exploitation. Safeguards against exploitation include avoiding a quid-pro-quo arrangement between service provision and study enrollment. All individuals who need intervention, not only those who fit the scientific inclusion criteria, should be treated. This has been a guiding principle for Project Prakash, which has treated not only congenitally blind children but also those with late-onset visual impairment. Also, independent institutional review boards must provide oversight to ensure participant welfare.
